# Supplementary material for: Loss of Cell-Cell Contact Inhibits Cellular Differentiation of α-Catenin Knock Out P19 Embryonal Carcinoma Cells and Their Colonization into the Developing Mouse Embryos
Source: BioTech (Basel). 2024 Oct 3;13(4):41. doi: 10.3390/biotech13040041 (PMC12456414; doi:10.3390/biotech13040041)
Supplement: Supplementary file 1 [file biotech-13-00041-s001.zip › biotech-3168675-supplementary.pdf]

BioTech

Supplementary material

Loss of Cell–Cell Contact Inhibits Cellular Differentiation of α-Catenin Knock Out P19 Embryonal Carcinoma Cells and Their Colonization into the Developing Mouse *Embryos*

Masahiro Sato, Emi Inada, Naoko Kubota and Masayuki Ozawa

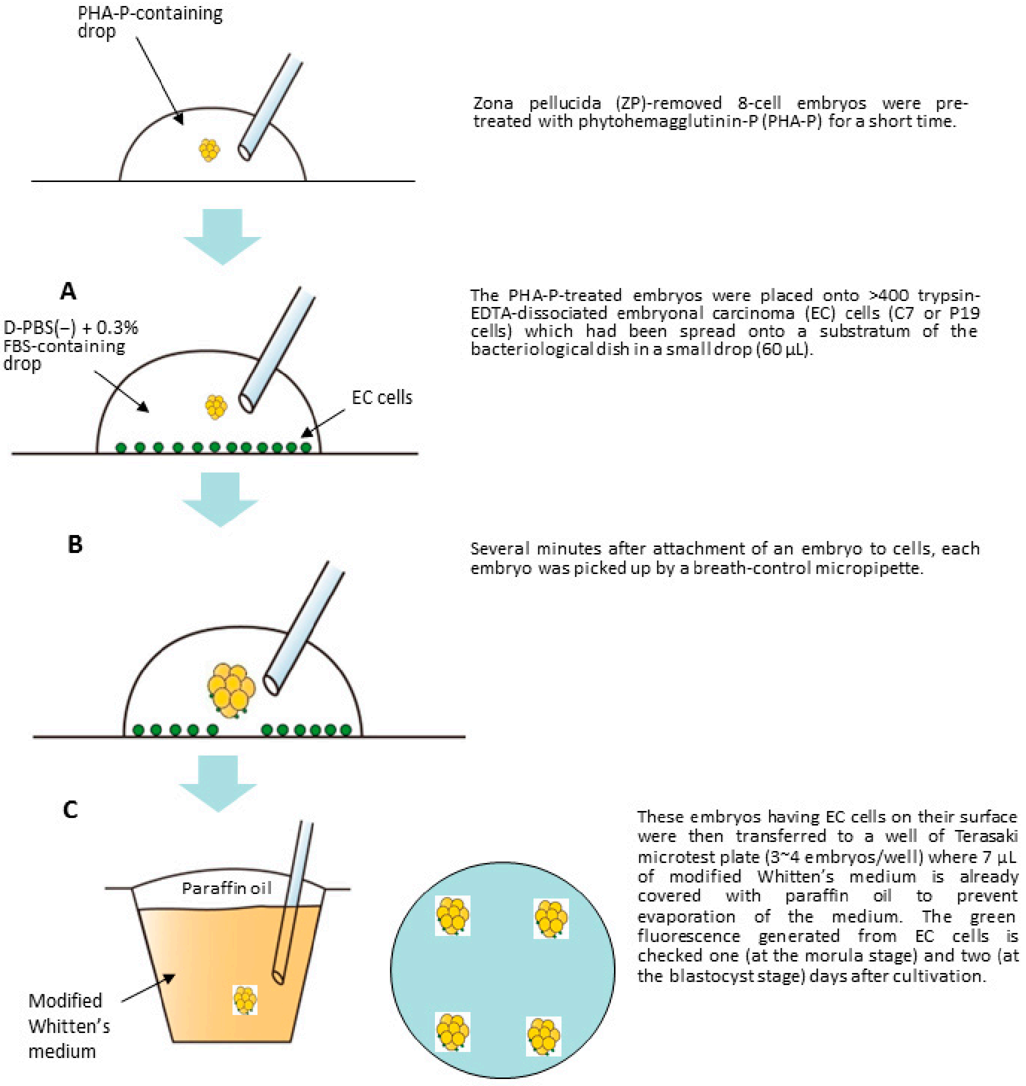

Figure S1. Generating aggregation chimeras between EC cells and embryos.
